# CARWL score as a predictor of radiation-induced periodontitis in locally advanced head and neck cancer undergoing concurrent chemoradiotherapy

**DOI:** 10.17305/bb.2025.13335

**Published:** 2025-11-20

**Authors:** Sibel Bascil, Efsun Somay, Nilüfer Kılıc Durankuş, Şükran Senyürek, Düriye Ozturk, Ugur Selek, Erkan Topkan

**Affiliations:** 1 Department of Periodontology, Faculty of Dentistry, Baskent University, Ankara, Türkiye; 2 Department of Oral and Maxillofacial Surgery, Faculty of Dentistry, Baskent University, Ankara, Türkiye; 3 Department of Radiation Oncology, School of Medicine, Koc University, Istanbul, Türkiye; 4 Department of Radiation Oncology, Faculty of Medicine, Afyonkarahisar Health Sciences University, Afyonkarahisar, Türkiye; 5 Department of Radiation Oncology, Faculty of Medicine, Baskent University, Adana, Türkiye

**Keywords:** C-reactive protein, serum albumin, significant weight loss, periodontitis, head and neck cancer, chemoradiotherapy

## Abstract

Although concurrent chemoradiotherapy (CCRT) has improved outcomes in locally advanced head and neck cancer (LA-HNC), radiation-induced periodontitis (RIP) remains an under-recognized oral toxicity with significant consequences, including tooth loss and osteoradionecrosis. This study evaluates the utility of the novel CARWL score—a combined index of the C-reactive protein-to-albumin ratio (CAR) and significant weight loss (SWL)—for stratifying the risk of RIP in LA-HNC patients without baseline periodontitis undergoing CCRT. We conducted a retrospective analysis of 67 LA-HNC patients who underwent CCRT and received detailed oral examinations before and after treatment; none had periodontitis at the initiation of CCRT. Receiver operating characteristic (ROC) curve analysis identified an optimal pretreatment CAR cutoff of 3.07, with SWL defined as greater than 5% body weight loss in the preceding six months. Based on CAR (≥3.07 vs <3.07) and SWL (present vs absent), patients were categorized into three CARWL groups. The primary endpoint was the association between the baseline CARWL group and the rates of RIP following CCRT. RIP was diagnosed in 17 patients (25.4%) during follow-up, with incidences increasing progressively across CARWL-0, CARWL-1, and CARWL-2 groups (11.8% vs 20.8% vs 38.5%; *P* ═ 0.007). In multivariable Cox proportional-hazards analysis, a higher CARWL score emerged as an independent predictor of increased RIP risk (adjusted HR = 3.64; 95% CI 1.41–9.37; *P* ═ 0.007), and supplementary logistic regression sensitivity analysis corroborated these findings (adjusted OR = 3.58; 95% CI 1.35–9.45). These findings demonstrate that the pretreatment CARWL score serves as a straightforward and readily available biomarker that effectively stratifies the risk of RIP in LA-HNC patients treated with CCRT.

## Introduction

The primary treatment modalities for locally advanced head and neck cancers (LA-HNCs) comprise organ-sparing definitive concurrent chemoradiotherapy (CCRT) and neoadjuvant or adjuvant radiotherapy (RT) with or without chemotherapy, tailored to the patient’s performance status and pathological risk factors [1, 2]. Advances in RT planning systems and delivery techniques have significantly enhanced survival rates and tumor control in these patients [3]. Nevertheless, despite these advancements, RT and CCRT are associated with numerous acute and chronic complications, particularly affecting the oral cavity [4–7]. Among the most severe complications are tooth caries, tooth loss, osteoradionecrosis, radiation-induced trismus, and severe radiation-induced periodontitis (RIP), all of which substantially diminish the quality of life for head and neck cancer (HNC) patients [8]. Unfortunately, RIP has garnered less attention compared to other radiation-induced toxicities, despite its potential for severe outcomes such as tooth loss and osteoradionecrosis.

Periodontitis is a widespread concern; however, it presents an especially significant risk for individuals undergoing RT in the head and neck region. These patients are more vulnerable to oral health issues, with RIP being a major concern [8]. Approximately 70% of patients experience increased periodontal attachment loss following RT [9, 10]. The effects of RT and CCRT lead to alterations in vascularity and cellularity of both soft and hard tissues, impairment of salivary gland function, and modifications to collagen synthesis [8]. These detrimental changes result in hypovascular, hypocellular, hypoxic, hyperinflated, and hyperfibrotic oral tissues, hindering the healing capacity of bone and soft tissues and elevating the risk of infections and necrosis [8, 11, 12]. RT modifies both the vascular and cellular composition of periodontal tissues, disrupting the synthesis of the periodontal ligament, misaligning existing Sharpey fibers, and enlarging the periodontal ligament space [13]. These radiation-induced tissue alterations enhance susceptibility to RIP and compromise the ability to regenerate and restore bone [14]. Additionally, RT or CCRT may exacerbate RIP by inducing oral dysbiosis, resulting in a shift from a healthy microbiome to pathogenic dominance. This dysbiotic oral environment increases the risk of plaque accumulation and loss of periodontal attachment, key factors that facilitate the onset of severe RIP [15]. In this context, the radiation dose, particularly the mean oral-cavity dose (MOCD), serves as a critical determinant of oral toxicity risk, reflecting the cumulative exposure of soft and hard tissues to therapeutic irradiation. Higher MOCD values have been associated with increased incidences of mucositis, xerostomia, and other oral complications, suggesting a potential link to the development of RIP.

To date, few studies have investigated the progression of RIP in HNC patients. Research by Marques and Dib [10] documented a significant reduction in periodontal attachment in irradiated areas 6–8 months post-RT compared to non-irradiated regions. Similarly, Schuurhuis et al. [14] observed an increase in periodontal pocket depth (with pockets deepening by 4–5 mm) and/or the emergence of new periodontal pockets of 4 mm or more following radiation therapy. Untreated periodontitis can lead to chronic inflammation and, ultimately, tooth loss [16]. The loss of teeth in HNC patients heightens the risk of malnutrition, weight loss (WL), and cachexia, consequently diminishing their chances of prolonged survival [17–19]. WL may significantly impact the prognosis of periodontal disease by impairing immune responses and treatment tolerance. In this context, Sales-Peres et al. [20] found a correlation between WL and increased gingival bleeding, peaking six months post-bariatric surgery.

Numerous inflammatory markers have been evaluated for prognostic classification and toxicity prediction in HNC patients, with C-reactive protein (CRP) and albumin (ALB) being among the most frequently studied. Chronic inflammation, such as that caused by periodontitis, results in a systemic increase in inflammatory cytokines, leading to elevated CRP levels [21]. Increases in CRP levels are consistently associated with decreased ALB levels, due to the inhibitory effects of CRP on ALB synthesis in hepatocytes. Furthermore, reduced ALB production during prolonged inflammation indicates a state of tissue catabolism due to nutrient deficiency, resulting in WL [18]. Kshirsagar et al. [22] investigated the relationship between periodontitis and serum levels of CRP and ALB, revealing a strong correlation between severe periodontitis and diminished serum ALB levels. Therefore, periodontitis can significantly impact CRP and ALB levels in the blood, with levels correlating to disease severity. However, in the absence of periodontitis, varying degrees of inflammation related to HNC and RT or CCRT may also contribute to the development of periodontitis, particularly in individuals receiving high radiation doses to their periodontal tissues [23]. Supporting this observation, Sakai et al. [24] reported that cancer patients exhibited significantly higher rates of periodontitis (81.0% vs 52.7%, *P* < 0.01) and severe periodontitis (44.0% vs 12.4%, *P* < 0.01) compared to individuals in a national survey.

A significant correlation exists between body weight and periodontitis. Periodontitis, a reflection of inadequate oral health, can result in WL. Conversely, involuntary WL or being underweight can significantly increase the risk of developing osteoporosis and tooth loss due to heightened susceptibility to periodontitis. In a comprehensive study involving a large Korean cohort, Song et al. [25] found that individuals with a BMI below 18.5 kg/m^2^, indicating underweight status, faced a markedly increased risk of periodontitis and tooth loss.

Recently, Topkan et al. introduced a new scoring system for assessing immune, inflammatory, and nutritional status, termed the CARWL scores. This system integrates the CRP-to-ALB ratio (CAR) and significant WL (SWL), defined as involuntary WL greater than 5% within the previous six months [26]. In their pioneering study, the authors demonstrated that this scoring system effectively stratified stage IIIC patients into three groups with significantly different survival outcomes, seemingly surpassing the efficacy of the current American Joint Committee on Cancer (AJCC) staging framework. However, despite its robust performance, the novel CARWL scoring system has not yet been evaluated for its predictive efficiency regarding RIP rates following RT or CCRT in HNC patients. Consequently, this retrospective cohort study aimed to assess the predictive ability of pretreatment CARWL scores for the development of RIP in patients with LA-HNC who exhibited no signs of periodontitis prior to CCRT.

## Materials and methods

### Study population, ethics, and consent

This retrospective cohort analysis adhered strictly to the principles outlined in the Declaration of Helsinki and its subsequent revisions. Prior to data collection, the research design (Project No. DKA19/39A) underwent a comprehensive review and received approval from the Institutional Assessment Board of Baskent University. All eligible patients provided written informed consent before initiating the prescribed therapy. This consent permitted the analysis of blood tests and pathology specimens and the dissemination of study findings through academic publications or conference presentations.

**Figure 1.**
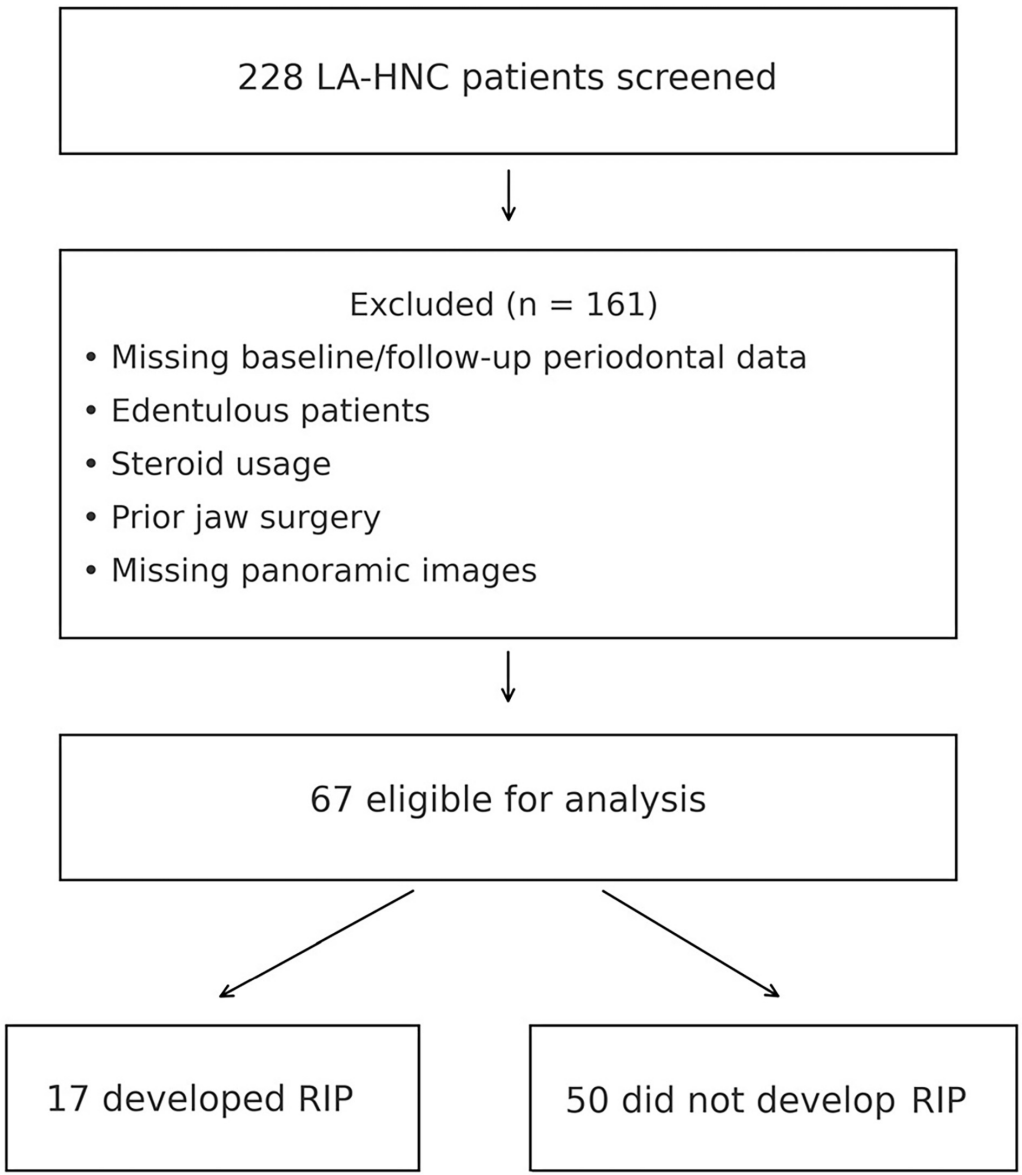
**Flowchart illustrating the patient selection process**. Abbreviations: LA-HNC: Locally advanced head and neck cancer; RIP: Radiation-induced periodontitis.

The Department of Radiation Oncology and the Dentistry Clinics at Baskent University’s Adana Research and Treatment Center collaborated in designing this research. A retrospective review of medical records was conducted for LA-HNC patients who received CCRT and underwent oral and dental examinations before and after treatment, spanning from February 2010 to January 2024 (Figure 1). Although both investigations were carried out at the same institution, the present dataset was compiled independently of our previously published 2024 cohort [27], and no patients from that series were included in this analysis. The current study population was restricted to patients with complete baseline and follow-up periodontal assessments. To ascertain the exact rates of RIP and their correlation with CARWL score groups, all participants were required to have documented evidence of no periodontitis prior to the initiation of CCRT. Eligibility criteria included: being at least 18 years of age, having histopathologic confirmation of squamous cell carcinoma, being classified as having locally advanced disease according to the 8th edition of the AJCC cancer staging criteria (T1-2N1-3M0 or T3-4N0-3M0), no prior history of other cancers, no prior systemic chemotherapy or RT in the HNC region, and availability of complete blood count and biochemistry test results before CCRT. Additional qualifications required access to dental and panoramic radiographic examination records before and after the completion of CCRT. Patients with a prior history of jaw surgery, documented tumor or lymph node invasion in the mandible or maxilla, or osteoradionecrosis of the jaws were excluded from the study. The research protocol also deemed patients on steroids or other immunosuppressive medications ineligible. To minimize the potential influence of preexisting inflammatory and immunological conditions and medication use on outcomes, individuals with chronic systemic immune or inflammatory diseases were also excluded from the analysis.

### Oral examinations and management

We conducted comprehensive oral and dental screenings for all patients, which included clinical and radiographic examinations. An experienced maxillofacial surgeon (ES) and a periodontist (SB) performed dental assessments prior to CCRT, adhering to the guidelines set forth by the American Dental Association (ADA) and the U.S. Food and Drug Administration (FDA) [28]. Each patient underwent radiographic examinations utilizing panoramic scans, following the manufacturer’s specifications (J. Morita, Veraviewepocs 2D, Kyoto, Japan). We employed illuminated mirrors and explorers to examine all teeth for dental caries, in accordance with World Health Organization guidelines [29].

The periodontal examination encompassed evaluations of plaque and gingival bleeding scores, probing depth (PD), mobility, and periodontal attachment loss. Plaque accumulation was assessed using the Silness–Löe Plaque Index on the buccal surfaces of all teeth. PD, bleeding on probing (BOP), and clinical attachment level (CAL) were documented at six sites per tooth (mesiobuccal, buccal, distobuccal, mesiolingual, lingual, distolingual) utilizing a UNC-15 periodontal probe with controlled insertion force not exceeding 2 mm. Whole-mouth periodontal scores for plaque, BOP, PD, and CAL were calculated as the mean of all measured sites, and the percentage of sites exhibiting BOP was also recorded. All periodontal examinations were conducted by a single, experienced periodontist, ensuring methodological consistency [30].

Two methods were employed to assess gingival inflammation. The Gingival Index was utilized to evaluate inflammation for each tooth [31], with scores ranging from 0 (normal gingiva) to 3 (severe inflammation). The Gingival Index scores were recorded for the buccal surface of each tooth. Additionally, gingival BOP was assessed; during examinations, all teeth were thoroughly evaluated on six surfaces. The score was determined by inserting a periodontal probe no more than 2 mm into the sulcus at the gingival border, specifically at the mesiobuccal line angle, and then advancing it along the buccal surface to the distobuccal line angle. The presence (1) or absence (0) of blood was documented after inspecting each tooth in a quadrant. The bleeding-on-probing score was calculated by summing the number of teeth with bleeding sites for each patient. PD and attachment loss assessments employed a UNC-15 periodontal probe at six designated areas on each tooth. Two measurements were recorded at each probing location: the distance from the free gingival margin to the cementoenamel junction, and the distance from the free gingival margin to the pocket base, with the pocket depth serving as the second measurement. Attachment loss was computed by subtracting the initial measurement from the second measurement when the free gingival margin was positioned above the cementoenamel junction, and by adding the two measurements when it was located below this junction.

The diagnosis of RIP disease adhered strictly to the 2018 AAP/EFP Classification of Periodontal and Peri-Implant Diseases and Conditions. Periodontitis was diagnosed based on: (i) CAL at two or more non-adjacent teeth or (ii) interdental CAL of ≥3 mm with PDs of ≥4 mm at two or more teeth, substantiated by radiographic evidence of alveolar bone loss. Staging and grading were performed according to the official consensus framework. Notably, dental caries, endodontic pathology (periapical lesions), and non-periodontal causes of tooth mobility were excluded from the case definition. References to Miller’s recession classification, which is a gingival recession index rather than a mobility scale, were removed. Consequently, the endpoint was restricted exclusively to periodontal disease. All RIP cases were re-evaluated and re-tabulated according to this standardized definition, yielding unchanged overall results [32, 33].

The periodontist underscored the importance of oral hygiene and provided self-care instructions for patients. Plaque and calculus were removed from patients with gingivitis to enhance oral hygiene and optimize oral health, and superficial tooth decay was addressed through fillings. Notably, no patient exhibited periodontitis at baseline, and the complete baseline periodontal status of all patients is detailed in Table S1.

### Assessment of CAR and SWL

For each patient, CRP and serum ALB levels were obtained from laboratory records on the first day of CCRT [27]. CRP was measured in mg/L using an immunoturbidimetric assay at an ISO 15189-accredited core laboratory (Baskent University), while ALB was recorded in g/dL and converted to g/L (multiplied by 10) prior to analysis. The CAR was calculated as CRP (mg/L) divided by ALB (g/L). Body weight was abstracted from clinic scale measurements whenever available. The weight target from 6 months pre-CCRT was set at --180 days (acceptable window: --195 to --165 days). If no clinic record was available within that interval, a patient-reported weight was documented and flagged. Percent WL was calculated using the formula:

%WL ═ [(Weight_--6___mo__ -- Weight-baseline) / Weight_--6___mo__] × 100,

where positive values indicate WL. A sensitivity analysis excluding self-reported weights yielded results consistent with the primary analysis. SWL was defined as a reduction in weight exceeding 5% during the previous 6 months, in accordance with the Delphi criteria established by Fearon et al. [34].

### Chemoradiotherapy protocol

In January 2010, the Department of Radiation Oncology adopted intensity-modulated radiotherapy (IMRT) as the standard treatment for patients with LA-HNC. The RT technique employed for all patients in this study was simultaneous integrated boost IMRT (SIB-IMRT) [35]. To enhance target volume delineation accuracy, co-registered computed tomography (CT), 18F-FDG PET/CT, and magnetic resonance imaging (MRI) datasets were routinely utilized. The oral cavity was contoured following the EORTC Head and Neck Cancer contouring guidelines [36], which included the mucosal surfaces of the anterior and posterior oral cavity—namely the buccal mucosa, gingiva, oral tongue (excluding the base), floor of mouth, and hard palate—while excluding the teeth, mandible, and maxilla. All contours underwent peer review as part of the institutional RT plan quality assurance process, and dose–volume histograms (DVHs) were exported directly from Eclipse for quantitative analysis. For analytical purposes, the MOCD was extracted from DVHs generated in the Eclipse Treatment Planning System (Varian Medical Systems, version 15.6).

The prescribed SIB-IMRT doses for the high-risk, intermediate-risk, and low-risk planning target volumes (PTVs) were 70 Gy, 59.4 Gy, and 54 Gy, respectively, delivered in 33 daily fractions over 5 days per week. In conjunction with IMRT, three cycles of concurrent cisplatin (80 mg/m^2^) were administered every 21 days. Following the completion of CCRT, all patients were advised to undergo two additional cycles of adjuvant chemotherapy with cisplatin and 5-fluorouracil. Antiemetic prophylaxis, nutritional supplementation, and other supportive care were provided in accordance with institutional protocols.

### Follow-up dental examination

The protocol outlined in the “baseline oral examination” section was adhered to, and subsequent oral and dental exams were conducted according to the established schedule or based on clinical indications. Clinical and radiological examination data were recorded at 1, 3, 6, 9, and 12 months post-CCRT, and every 6 months thereafter throughout the follow-up period. Treatment criteria for each patient were established and presented according to the concepts outlined in the aforementioned “baseline oral examination” section.

**Table 1:** Periodontitis rates according to baseline and treatment characteristics

**Characteristics**	**All patients (*n* ═ 67)**	**Periodontitis *n* ═ 17 (%)**	**Univariate *P* value**	**Multivariate *P* value**	**Multivariate HR (95% CI)**
Median age group					
<56 years ≥56 years	36 31	11 (30.5) 6 (19.4)	0.40	–	–
Gender					
Female Male	20 47	4 (20.0) 13 (27.7)	0.76	–	–
Smoking status					
Yes No	40 27	13 (32.5) 4 (14.8)	0.011	0.023	2.07 (1.42–4.08)
Alcohol consumption status					
Yes No	24 43	6 (25.0) 11 (25.6)	1.0	–	–
Type of cancer, *N* (%)					
Oral cavity Nasopharynx	34 33	6 (26.1) 8 (24.2)	0.79	–	–
T-stage group					
1-2 3-4	29 38	4 (13.8) 13 (34.2)	0.014	0.021	2.27 (1.52–3.84)
N-stage group					
0-1 2-3	25 42	5 (20.0) 12 (28.5)	0.26	–	–
Concurrent chemotherapy cycles					
1 2-3	15 52	2 (13.3) 15 (28.8)	0.008	0.013	2.48 (1.65–4.03)
Adjuvant chemotherapy cycles					
0 1-2	25 42	5 (24.0) 11 (26.2)	0.39	–	–
MOCD group, *N* (%)					
≤50.1 Gy >50.1 Gy	36 31	5 (13.8) 12 (38.7)	<0.001	<0.001	2.98 (1.94--5.67)
CARWL group					
CARWL-0 (Reference) CARWL-1 CARWL-2	17 24 26	2 (11.8) 5 (20.8) 10 (38.5)	0.007	0.007	– 1.78 (1.22--3.68) 3.64 (1.41--9.37)

RIP rates and 95% confidence intervals are determined using exact Clopper–Pearson estimates. Comparisons among the CARWL subgroups (CARWL-0, CARWL-1, and CARWL-2) were adjusted for multiple testing through the Bonferroni method, resulting in an adjusted significance threshold of α-adj = 0.0167 (0.05/3). Abbreviations: CCRT: Concurrent chemoradiotherapy; T: Tumor; N: Node; Gy: Gray; MOCD: Mean oral cavity dose.

### Statistical analysis

The primary objective of this study was to evaluate the association between pretreatment CARWL scores and the time to development of RIP during follow-up after CCRT in LA-HNC patients without baseline periodontitis. Continuous variables were summarized as medians (range), while categorical variables were expressed as frequency percentages. Intergroup differences were assessed using the chi-square test, Student’s *t*-test, or Spearman correlation, as appropriate. Receiver operating characteristic (ROC) analysis was employed initially to determine an optimal pretreatment CAR cutoff (Youden’s J statistic) for stratifying the cohort by outcome risk. Given that RIP could occur at varying follow-up times, the primary analysis utilized a Cox proportional-hazards model, with all effect measures reported as hazard ratios (HRs) and corresponding 95% confidence intervals (CIs). The proportional-hazards assumption was verified using Schoenfeld residuals. To explore potential nonlinearity in the CAR-RIP relationship, restricted cubic splines (three knots at the 10th, 50th, and 90th percentiles) were incorporated into the Cox model, with the *P* value for nonlinearity guiding interpretation. To confirm the robustness and directionality of associations, a secondary logistic-regression sensitivity model was fitted, with effect estimates reported as odds ratios (ORs) and 95% CIs. Due to the limited number of RIP events (*n* ═ 17), ridge-penalized Cox regression was applied to mitigate overfitting and small-sample bias. Candidate covariates were prespecified based on clinical relevance: age, sex, T category, N category, smoking status, MOCD, number of concurrent chemotherapy cycles, baseline periodontal status, and CARWL group. Variance inflation factors (VIFs) were calculated to evaluate potential multicollinearity among predictors, with all values <2.0 indicating acceptable independence among covariates. Additionally, a MOCD×CARWL interaction term was tested; however, it did not reach statistical significance and was therefore excluded from the final Cox model. Internal validation was conducted using 1000 bootstrap resampling to estimate bias-corrected C-statistics (Harrell’s C), calibration intercepts, and slopes, accompanied by calibration plots. The optimism-corrected model performance, including that of the continuous CAR spline model, was derived from this bootstrap procedure. All statistical tests were two-tailed, with a significance level of *P* < 0.05 considered statistically significant. To account for potential inflation of type I error from multiple subgroup comparisons, the Bonferroni correction was applied exclusively to analyses involving the three-level CARWL classification (CARWL-0, CARWL-1, and CARWL-2). As these strata yielded three pairwise contrasts, the adjusted significance level was set at α-adj = 0.05/3 = 0.0167. All Bonferroni-adjusted *P* values (p-adj) are reported in tables and figures, with footnotes specifying the correction method.

### Ethics approval and consent to participate

Prior to data collection, the study protocol received approval from the Institutional Review Board of the Baskent University School of Medicine and adhered to the principles outlined in the Declaration of Helsinki.

## Results

This retrospective study analyzed data from 228 patients diagnosed with LA-HNC who underwent CCRT. Of these, 67 patients met the inclusion criteria, while 161 were excluded due to being edentulous or lacking pre- and postoperative dental or periodontal records. As presented in Table 1, the study population consisted of 50.7% with oral cavity cancer and 49.3% with nasopharyngeal cancer. The cohort included 70.1% men, with a median age of 56 years (range, 18–75). A history of smoking and alcohol consumption was reported in 59.7% and 35.8% of patients, respectively. A significant proportion of patients presented with advanced primary (56.8%) or nodal (62.7%) disease.

CCRT was generally well tolerated, with 24 cases (35.8%) of grade 3 mucositis and four cases (6.0%) of grade 4 mucositis, and no treatment-related fatalities. The median duration of CCRT was 47 days (range, 45–55 days). Five patients (8.2%) experienced brief treatment interruptions (≤5 days) due to grade ≥3 radiation-induced mucositis, after which therapy was resumed as scheduled. During the CCRT and adjuvant phases, 77.6% and 62.7% of patients completed the planned 2–3 and 1–2 cycles of chemotherapy, respectively. RT target volumes included the primary tumor and elective nodal regions, as delineated by institutional contouring protocols. Among the 67 patients, 58 (86.6%) received radiation targeting level Ib, while 61 (91.0%) had coverage extending to level IIb nodal regions. The MOCD was 50.1 Gy (range, 10.8–61.2 Gy), with 53.7% receiving doses ≥50.1 Gy. The median mandibular dose was 38.4 Gy (range, 11.8–62.4 Gy), with 47.8% receiving doses exceeding 38.4 Gy. At a median follow-up of 69.6 months (range, 7.8–146.9 months), 37 patients (55.2%) underwent 1–3 tooth extractions, and 17 (25.4%) developed RIP at a median of 10.0 months (range, 5.3–17.1 months). RIP was absent in 74.6% of cases and present in 25.4%.

**Figure 2.**
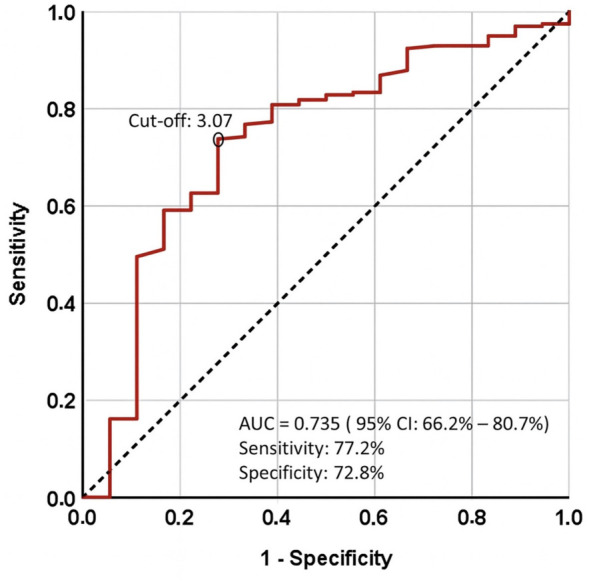
**Receiver operating characteristic (ROC) curve of CAR for prediction of RIP.** ROC curve analysis identified an optimal CAR cutoff for RIP at 3.07, with an AUC of 73.5%, sensitivity of 77.2%, specificity of 72.8%, and a J-index of 0.50. RIP rates and their corresponding 95% confidence intervals were calculated using exact (Clopper–Pearson) methods. Comparisons among CARWL subgroups (CARWL-0, CARWL-1, and CARWL-2) were adjusted for multiple testing through the Bonferroni method, resulting in an adjusted significance threshold of α-adj = 0.0167 (0.05/3). Abbreviations: CAR: C-reactive protein-albumin ratio; RIP: Radiation-induced periodontitis; AUC: Area under the curve; Sens: Sensitivity; Spec: Specificity.

ROC curve analysis determined an optimal cutoff for the CAR of 3.07 for predicting RIP (AUC = 0.735; sensitivity = 77.2%; specificity = 72.8%; Youden’s *J* ═ 0.50) (Figure 2). In accordance with the Delphi consensus by Fearon et al. [34] and the CARWL framework by Topkan et al. [26], SWL was defined as a loss of ≥5% body weight within 6 months prior to CCRT. Based on these parameters, patients were stratified into four CARWL categories: Group 1 (CAR < 3.07 and WL ≤ 5%), Group 2 (CAR < 3.07 and WL > 5%), Group 3 (CAR ≥ 3.07 and WL ≤ 5%), and Group 4 (CAR ≥ 3.07 and WL > 5%). The corresponding incidences of RIP were 11.8% (95% CI: 5.2–24.9%), 21.4% (95% CI: 8.3–43.0%), 22.7% (95% CI: 9.9–45.2%), and 38.5% (95% CI: 20.2–61.4%), respectively. Due to statistically indistinguishable rates in Groups 2 and 3 (χ^2^ ═ 0.18, *P* ═ 0.67) with overlapping CIs, these groups were merged to form a three-tier CARWL schema: CARWL-0 (CAR < 3.07 and WL ≤ 5%), CARWL-1 (CAR < 3.07 and WL > 5% or CAR ≥ 3.07 and WL ≤ 5%), and CARWL-2 (CAR ≥ 3.07 and WL > 5%).

**Figure 3.**
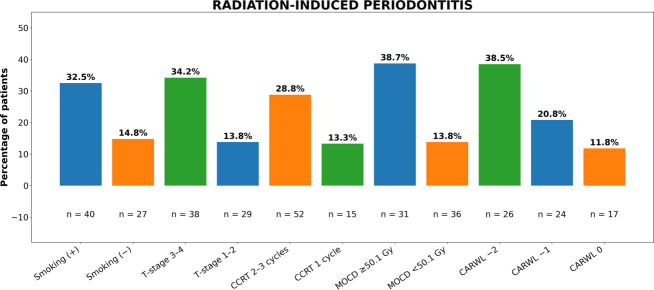
**Rates of radiation-induced periodontitis by contributing factors.** Bar plots illustrate the proportion of patients who developed RIP based on smoking status, T stage, number of CCRT cycles, MOCD, and CARWL category. The incidence of RIP exhibited a progressive increase across CARWL strata (11.8%, 20.8%, 38.5%; likelihood-ratio trend test *P* ═ 0.006; omnibus Wald *P* ═ 0.007), thereby reinforcing the ordered structure of the CARWL index. Abbreviations: T: Tumor; CCRT: Concurrent chemoradiotherapy; MOCD: Mean oral cavity dose; Gy: Gray; CARWL: C-reactive protein to albumin ratio; WL: Weight loss.

Analysis of CARWL as an ordinal variable revealed a significant monotonic association with increasing RIP incidence (likelihood-ratio trend test *P* ═ 0.006), confirming the validity of its ordered structure. The incidence of RIP progressively increased across CARWL categories: 11.8% vs 20.8% vs 38.5% (*P* ═ 0.007, omnibus Wald test), indicating nearly doubled risk with each successive category (Figure 3, Table 2). In multivariable Cox proportional hazards analysis, adjusted HRs for RIP were 1.78 (1.22–3.68) for CARWL-1 and 3.64 (95% CI: 1.41–9.37) for CARWL-2, relative to CARWL-0. The logistic regression sensitivity model yielded comparable adjusted ORs: 1.82 (95% CI: 1.32–3.84) and 3.58 (95% CI: 1.35–9.45), respectively. For clinical interpretability, adjusted absolute risk differences derived from logistic marginal effects corresponded to +8.9% (95% CI: –4.2% to +19.3%) for CARWL-1 and +23.5% (95% CI: +8.6% to +37.8%) for CARWL-2 compared with CARWL-0. All VIFs were <2.0, confirming the absence of problematic multicollinearity among predictors. The interaction term MOCD×CARWL was not statistically significant (*P* ═ 0.53) and was excluded from the final multivariable model.

**Table 2:** The results of univariate and multivariate analysis depicting the significance level factors of radiation-induced periodontitis

**Characteristics**	**All patients (*n* ═ 67)**	**CARWL-0 (*n* ═ 17)**	**CARWL-1 (*n* ═ 24)**	**CARWL-2 (*n* ═ 26)**	***P* value**
Median age, years (range)	56 (18–75)	58 (43–75)	53 (22–72)	56 (18–69)	0.29
Median age group, years, *N* (%) ≥56 <56	36 (53.7) 31 (46.3)	12 (70.6) 5 (29.4)	9 (37.5) 15 (62.5)	15 (57.7) 11 (42.3)	0.98
Gender, *N* (%) Female Male	20 (29.9) 47 (70.1)	5 (29.4) 12 (70.6)	8 (33.3) 16 (66.7)	7 (26.9) 19 (73.1)	0.89
Smoking status, *N* (%) Yes No	40 (59.7) 27 (40.3)	10 (58.8) 7 (41.2)	14 (58.3) 10 (41.7)	16 (61.5) 10 (38.5)	0.97
Alcohol consumption status, *N* (%) Yes No	24 (35.8) 43 (64.2)	7 (41.2) 10 (58.8)	7 (29.2) 17 (70.8)	10 (38.5) 16 (61.5)	0.69
Median number of pre-CCRT tooth extraction, *N*, (range)	2 (0–10)	2 (0–10)	2 (0–4)	2 (0–7)	0.59
Type of cancer, *N* (%) Oral cavity Nasopharynx	34 (50.7) 33 (49.3)	7 (41.1) 10 (58.9)	14 (58.3) 10 (41.7)	13 (50.0) 13 (50.0)	0.34
T-stage group, *N* (%) 1-2 3-4	29 (43.2) 38 (56.8)	8 (47.1) 9 (52.9)	10 (41.7) 14 (58.3)	11 (42.3) 15 (57.7)	0.78
N-stage group, *N* (%) 0-1 2-3	25 (37.3) 52 (62.7)	7 (41.2) 10 (58.8)	9 (37.5) 15 (62.5)	9 (34.6) 17 (65.4)	0.63
Concurrent chemotherapy cycles, *N* (%) 1 2-3	15 (22.4) 52 (77.6)	3 (20.0) 11 (80.0)	5 (20.8) 19 (79.2)	7 (26.9) 20 (73.1)	0.57
Adjuvant chemotherapy cycles, *N* (%) 0 1-2	25 (37.3) 42 (62.7)	6 (35.2) 11 (64.8)	9 (37.5) 15 (62.5)	10 (38.5) 16 (61.5)	0.81
MOCD, Gy (range)	50.1 (10.8–61.2)	51.7 (13.4–60.2)	48.6 (10.8–59.3)	49.4 (11.4–61.2)	0.51
MOCD group, *N* (%) ≤50.1 Gy >50.1 Gy	36 (53.7) 31 (46.3)	9 (52.9) 8 (47.1)	12 (50.0) 12 (50.0)	15 (57.7) 11 (42.3)	0.42
Median post-CCRT extracted tooth, *N* (range)	1 (0-3)	0 (0-1)	1 (0-3)	1 (0-2)	0.79
Post-CCRT tooth extraction, *N* (%) Absent Present	30 (44.8) 37 (55.2)	9 (52.9) 8 (47.1)	9 (37.5) 15 (62.5)	12 (46.2) 14 (53.8)	0.61
RIP status, *N* (%) Absent Present	50 (74.6) 17 (25.4)	15 (88.2) 2 (11.8)	19 (79.2) 5 (20.8)	16 (61.5) 10 (38.5)	0.004
Median time from CCRT to periodontitis, mo	10.0 (5.3–17.1)	13.2 (10.4–17.1)	11.6 (11.2–14.1)	8.7 (5.3–11.4)	0.28

Abbrevations: CCRT: Concurrent chemoradiotherapy; T: Tumor; N: Node; MOCD: Mean oral cavity dose; Gy: Gray; RIP: Radiation-induced periodontitis; mo: Month.

Modeling CAR as a continuous variable with restricted cubic splines confirmed an approximately linear relationship with RIP hazard (*P* ═ 0.21 for nonlinearity). The optimism-corrected Harrell’s C-statistic (0.72) and calibration slope (0.94) indicated good discrimination and minimal overfitting following 1000 bootstrap validations. Collectively, these model-based analyses confirm that the risk and hazard of developing RIP increase in a graded, near-linear fashion across ascending CARWL categories, underscoring the prognostic significance of the CARWL index in this population.

**Figure S1.**
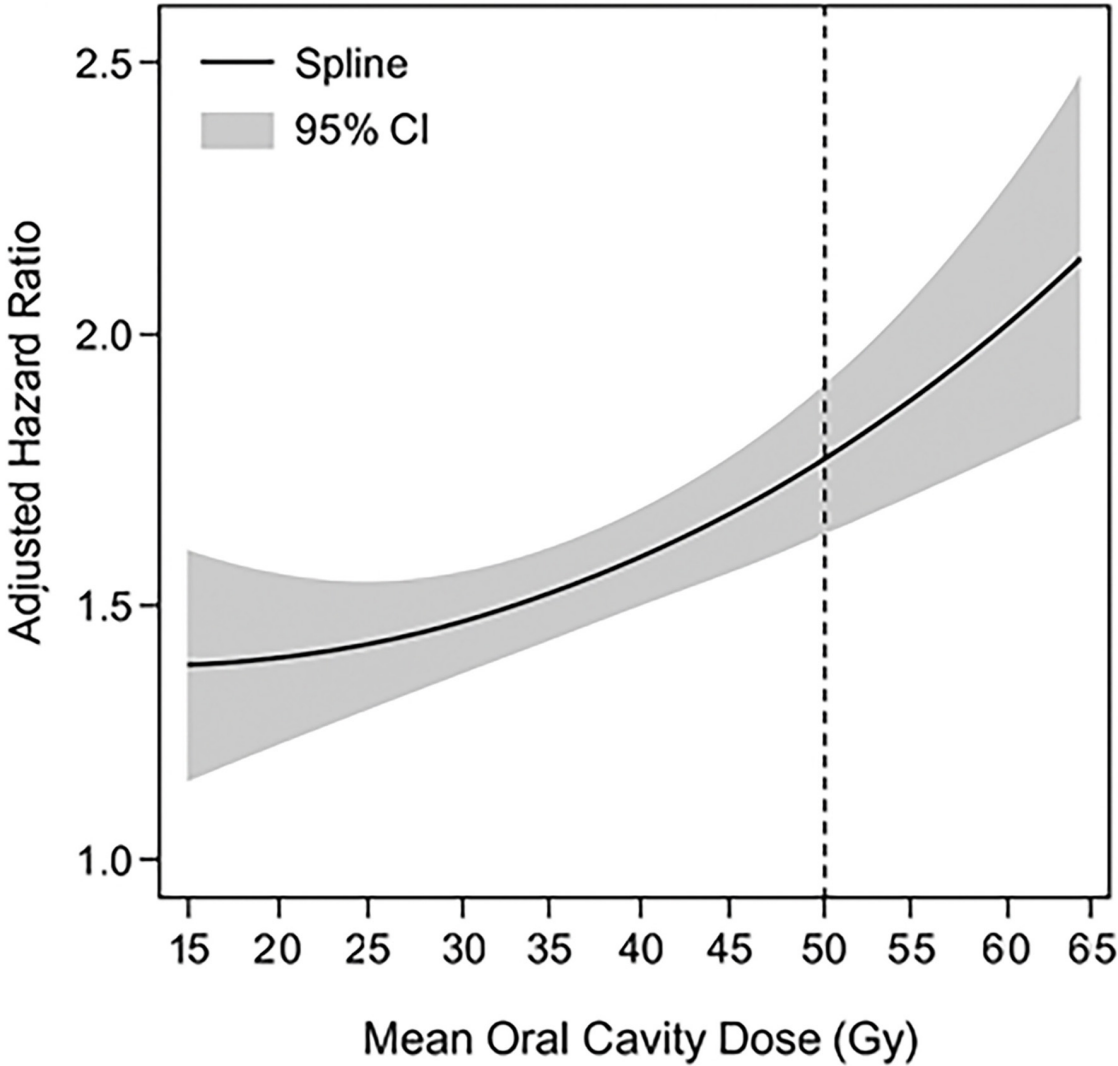
**Dose–response relationship between mean oral cavity dose and risk of radiation-induced periodontitis.** This restricted cubic spline analysis illustrates the correlation between mean oral cavity dose (Gy) and the adjusted hazard ratio for radiation-induced periodontitis (RIP), as derived from the Cox proportional hazards model. The solid line represents the estimated hazard ratio, while the shaded area indicates the 95% confidence interval. The curve demonstrates a monotonic and nearly linear increase in RIP risk with greater dose exposure (*P* for nonlinearity = 0.18). The vertical dashed line at 50.1 Gy signifies the clinically relevant cutoff point utilized in categorical analyses.

Given the robust association between MOCD > 50.1 Gy and the occurrence of RIP, additional analyses were conducted to assess this relationship as a continuous variable and to evaluate potential nonlinearity. When modeled continuously within the Cox proportional hazards framework, MOCD demonstrated a consistent, near-linear increase in RIP hazard, without significant evidence of nonlinearity (*P* for nonlinearity = 0.18). Each incremental increase of 1 Gy in MOCD was associated with an adjusted HR of 1.09 (95% CI: 1.03–1.17, *P* ═ 0.004) for developing RIP, confirming a dose-dependent relationship. The spline-based dose–response curve (Figure S1) illustrated a gradual, monotonic rise in risk across the dose range. For clinical interpretability and to align with existing literature, MOCD was dichotomized at 50.1 Gy in Table 2; however, the continuous-model results reinforce that the risk of RIP increases proportionally with higher doses of radiation exposure to the oral cavity.

**Figure S2.**
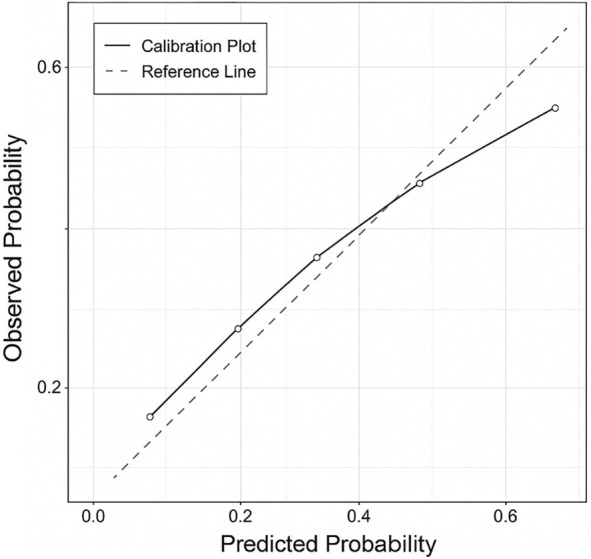
**Calibration of the multivariable Cox proportional-hazards model for radiation-induced periodontitis.** This calibration plot illustrates the agreement between predicted and observed probabilities of radiation-induced periodontitis derived from the final multivariable model. The diagonal dashed line represents perfect calibration, while the solid line indicates the bias-corrected performance of the model following 1000 bootstrap resamples. The model exhibited strong overall calibration, with minimal deviation at the extremes (C-index = 0.72; 95% CI: 0.64–0.80; calibration slope = 0.94; intercept ═ −0.03).

**Figure S3.**
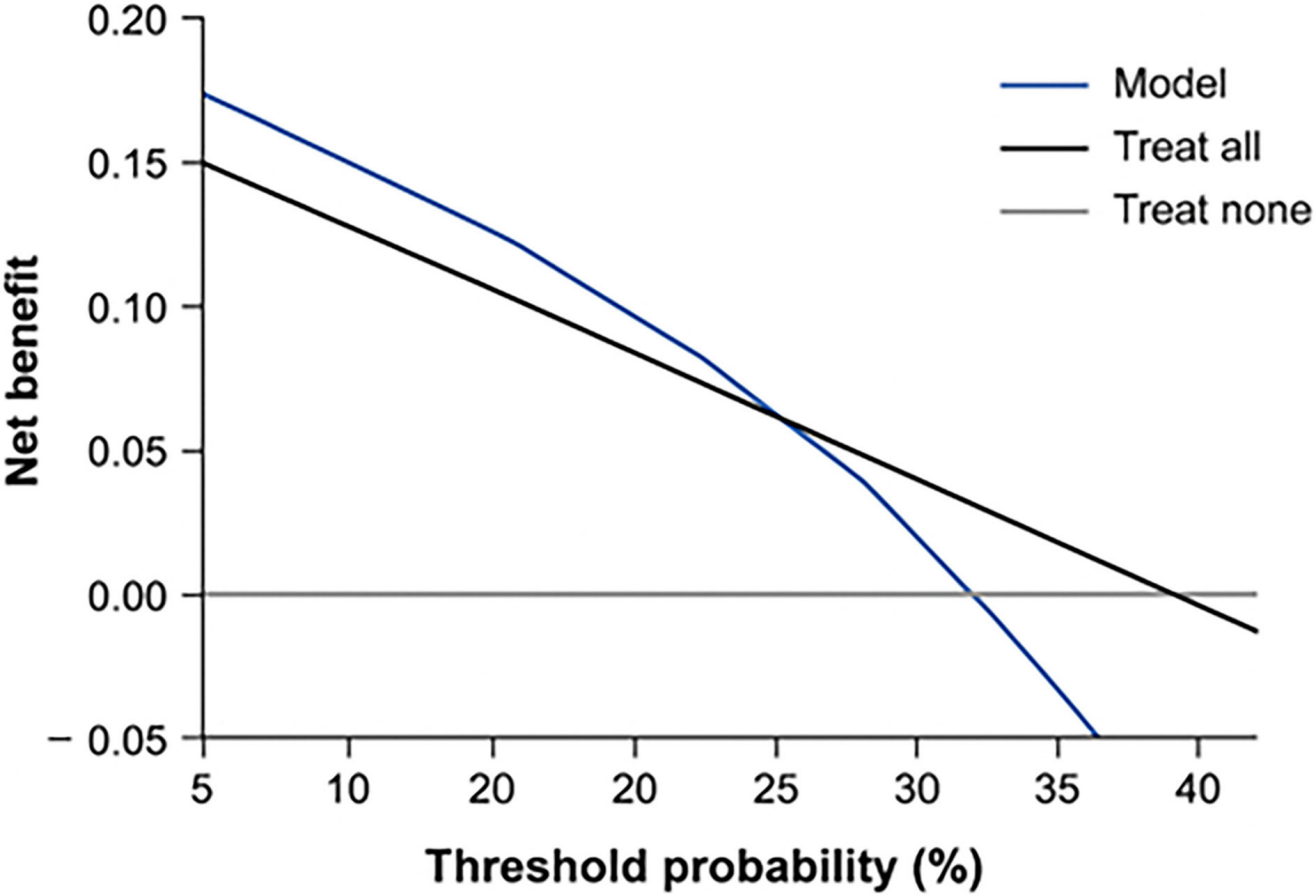
**Decision-curve analysis (DCA) for predicting radiation-induced periodontitis risk.** This DCA evaluates the net clinical benefit of the comprehensive multivariable model (solid blue line) relative to the treat-all (black line) and treat-none (gray line) strategies across threshold probabilities ranging from 5% to 40%. The comprehensive model demonstrated a consistently higher net benefit within the clinically relevant threshold range, indicating its potential utility for individualized risk prediction of radiation-induced periodontitis.

The final multivariable Cox model exhibited strong discrimination, achieving a Harrell’s C-index of 0.72 (95% CI: 0.64–0.80) through 1000 bootstrap resamples. Calibration analysis indicated a close alignment between predicted and observed risks, with a calibration slope of 0.94 and an intercept of --0.03, reflecting minimal overfitting. The calibration plot (Figure S2) confirmed the model’s robust performance across the entire spectrum of predicted probabilities. To evaluate clinical applicability, decision curve analysis (DCA) was conducted across threshold probabilities ranging from 5% to 40%, revealing a distinct net benefit of the comprehensive model compared to treat-all or treat-none strategies (Figure S3).

Univariate analyses identified significant predictors of RIP including smoking (*P* ═ 0.011), T3–4 stage (*P* ═ 0.014), receiving two or more chemotherapy cycles (*P* ═ 0.008), MOCD ≥ 50.1 Gy (*P* < 0.001), and a higher CARWL group (*P* ═ 0.007). In the final multivariable Cox model, the CARWL score, T3–4 stage, MOCD ≥ 50.1 Gy, and smoking each maintained independent significance (Table 2).

## Discussion

Periodontitis represents a significant oral health concern, often leading to tooth loss if untreated. This condition is particularly relevant for patients with LA-HNC undergoing CCRT, as it profoundly impacts their quality of life. Motivated by this issue, we aimed to investigate the effect of the pretreatment CARWL index on the risk of periodontitis following radiation therapy, a first in the literature. Our findings indicated that the RIP rate escalated significantly from CARWL-0 to CARWL-1 and CARWL-2 (11.8% vs 20.8% vs 38.5%; *P* ═ 0.007). Additionally, we identified statistically significant associations between RIP and smoking status (*P* ═ 0.023), T3-4 stage (*P* ═ 0.021), receiving 2–3 cycles of concurrent chemotherapy (*P* ═ 0.013), and MOCD > 50.1 Gy (*P* < 0.001).

Smoking is a critical factor with a notable impact on periodontal health [37]. Our study corroborated existing literature, finding a significant association between RIP and smoking (*P* ═ 0.023). Beyond the inflammatory effects of CCRT, smoking adversely impacts the vascularization of gingival tissues, hindering inflammatory and immune responses as well as the healing capacity of periodontal tissues. Furthermore, immune and inflammatory cells generate a variety of inflammatory mediators in response to smoking, with studies indicating elevated levels of inflammatory biomarkers such as CRP and IL-6 in the plasma of smokers compared to non-smokers [38]. The heightened risk of periodontal disease in patients subjected to head and neck radiation is generally linked to hyposalivation and alterations in the oral microbiome. Microscopic evaluations have revealed a dose-dependent loss of proliferative capacity in oral keratinocytes, alongside increased pro-inflammatory cytokine levels [13]. For instance, a review conducted by Irie et al. [39] analyzed 37 scientific articles discussing crucial aspects of periodontal treatment before and after radiation therapy in HNC patients, confirming that periodontal health deteriorates as a result of radiation, with tooth loss and advanced periodontal disease correlating with poor oral health prior to treatment. These findings, coupled with the established detrimental effects of smoking, highlight the necessity of thorough oral health assessments prior to radiation therapy in cancer patients, particularly those with a smoking history.

Another significant finding from our study is that the risk of RIP increases in patients with advanced T stages (T3-4) and those receiving 2–3 cycles of CCRT (*P* ═ 0.021 and *P* ═ 0.013, respectively). Standard treatment for patients with locally advanced stage III and IV tumors typically involves surgery with reconstruction followed by postoperative RT. When high-risk features are identified during surgery, treatment is often intensified through the addition of postoperative chemoradiotherapy to enhance outcomes and reduce recurrence risk. In cases of recurrence. In HNCs, the radiation dose generally correlates with tumor stage; as the stage increases, indicative of a more aggressive tumor, the radiation dose and treatment intensity are typically elevated to ensure optimal control [40]. Elevated radiation doses to the jawbone and oral tissues during HNC treatment significantly increase the risk of oral complications, including periodontitis [8]. During chemotherapy, agents can contribute to conditions such as mucositis, xerostomia, gingival bleeding, and periodontitis [41]. The severity of these issues is influenced by factors such as cancer type, chemotherapy regimen, number of cycles, and intervals between cycles [42]. Literature suggests that chemotherapeutic agents may directly affect the buccal mucosa through systemic circulation or indirectly through alterations in saliva secretion [41]. Furthermore, these agents can quantitatively and qualitatively disrupt salivary flow and its components, such as amylase and immunoglobulin A (IgA) [43]. For example, Azher and Shiggaon [44] assessed the oral health of children with acute lymphoblastic leukemia undergoing chemotherapy, finding that gingival inflammation peaked during the maintenance phase, followed by the induction phase with radiation therapy. Moreover, increasing radiation doses and the number of chemotherapy cycles in advanced-stage patients appear to exacerbate periodontal tissue damage, leading to heightened susceptibility to RIP following definitive CCRT. These findings suggest a potential additive detrimental effect of CCRT and cumulative chemotherapy exposure on periodontal structures.

In addition to treatment-related factors, patient-specific biological and behavioral aspects—particularly baseline oral hygiene and pre-treatment dental care—may influence periodontal vulnerability during CCRT. Despite all patients receiving standardized professional cleaning before therapy, individual differences in plaque control, gingival inflammation, and oral microbiome composition could modulate the host’s immune-inflammatory response. Poor plaque control is known to exacerbate neutrophil-mediated soft-tissue damage, disrupt cytokine expression (e.g., IL-1β and TNF-α), and promote a dysbiotic microbial community that is more susceptible to radiation-induced mucosal and periodontal breakdown. Variations in pre-treatment periodontal stability may act as confounders, altering the biological thresholds at which radiation and chemotherapy induce connective tissue destruction and alveolar bone loss. Recognizing these patient-specific factors is crucial for understanding RIP risk and for developing personalized preventive strategies prior to CCRT.

One of the primary findings of our study was the distinct relationship between the MOCD and the development of RIP, which aligns with existing literature. Although the majority of RIP events occurred in patients receiving an MOCD of ≥50.1 Gy, a few cases were observed below this threshold, indicating a dose-dependent—albeit not absolute—risk relationship. In our cohort, RIP was predominantly detected among patients who received MOCDs of ≥50.1 Gy, with a median onset time of 10 months post-CCRT (37.8% vs 13.8% for MOCD < 50.1 Gy, *P* < 0.001). RT exerts cytotoxic effects on both normal and malignant tissues, with direct damage to the oral mucosa, gingiva, and alveolar bone frequently accompanying CCRT. Indirect injury may also arise from systemic toxicity, inflammation, or vascular compromise due to treatment [45]. Supporting these findings, Hommez et al. [46] reported that teeth with apical periodontitis received significantly higher radiation doses than those with normal periapical status (37.2 Gy vs 24.9 Gy; *t* ═ 2.823, *P* < 0.01). Similarly, Pathomburi et al. [47] demonstrated that periodontal ligament cell proliferation decreased threefold when the local radiation dose exceeded 42 Gy compared to 20 Gy. Mechanistically, irradiation-induced vascular injury initiates a cascade of swelling, capillary degeneration, and necrosis, leading to increased permeability and progressive perivascular fibrosis [48]. The accumulation of fibrotic tissue ultimately results in capillary stenosis and obliteration, causing reduced vascularity and cellularity of the periodontal ligament, widening of the periodontal space, and thickening or distortion of Sharpey’s fibers. Over time, these microstructural alterations contribute to late-onset RIP and the deterioration of periodontal integrity [49].

The most significant result of our study is the substantial effect of the pretreatment CARWL index on the risk of severe periodontitis following RT. We demonstrated that the RIP rate significantly increased from CARWL-0 to CARWL-1 and CARWL-2 (11.8% vs 20.8% vs 38.5%; *P* ═ 0.007). While prior studies have highlighted the effects of the pre-CCRT systemic inflammation index (SIS) [50] and the GLUCAR index on tooth loss after CCRT [27], the influence of any inflammatory mediator on the RIP rate has not been previously established. Therefore, although it is challenging to compare our results with those of other studies, a better understanding of the mechanism will emerge when the components of the CARWL index are examined individually. Notably, CRP, a component of the CARWL index, is a pentameric plasma protein that plays a role in the systemic response to inflammation [51]. It is regulated by cytokines such as interleukin-6 (IL-6), interleukin-1β (IL-1β), and tumor necrosis factor-α (TNF-α) [52]. Furthermore, CRP may reflect changes in the cellular and molecular components of peripheral blood due to inflammatory conditions in individuals with periodontitis. Numerous studies have demonstrated a positive correlation between chronic periodontitis and elevated serum CRP levels [53, 54], as it is biologically plausible that inflammatory mediators (IL-1, IL-6, and TNF-α) released during periodontitis can stimulate hepatocytes to produce CRP. Consequently, it is expected that, in the presence of chronic periodontitis, higher serum CRP levels would be observed [51]. Conversely, Kuar et al. investigated the impact of another factor, ALB, on chronic periodontitis in 60 patients. They found that chronic periodontitis was more prevalent in the group with serum ALB levels below 4.815 g/dL (*P* < 0.001), attributing this to the association between low serum ALB levels and periodontal attachment loss [55]. Similarly, Ogawa et al. [56] reported an inverse independent association between periodontal disease and serum ALB concentrations. The final component, significant WL (%), indicative of malnutrition, has also been associated with periodontal tissue health; for instance, nutritional deficiencies have been linked to more rapid tissue deterioration. Dietary abnormalities tend to exacerbate the inflammatory processes involved in periodontal disorders [57]. Furthermore, malnutrition negatively impacts the development of the oral cavity and the progression of oral diseases by altering tissue homeostasis, decreasing resistance to microbial biofilms, and impairing tissue repair capacity [58]. Taken together, the differences observed between CARWL-0 and CARWL-1 and CARWL-2 in our study (11.8% vs 20.8% vs 38.5%; *P* ═ 0.007) align with expectations. Given that only one patient (1.5%) died prior to experiencing RIP, the number of competing events was too small to materially distort the estimated risk of RIP over time. Under these circumstances, any bias introduced by treating these deaths as non-informative censoring is expected to be minimal, allowing standard Kaplan–Meier estimation and Cox regression to provide risk estimates that are effectively equivalent to those obtained from formal competing-risk methods.

Several limitations constrain the present study. First, it relied on retrospective data from a single institution and included a relatively small sample, which may introduce unintentional selection bias—a common issue in such analyses. The limited cohort size is critical; the observed incidence of RIP was 25.4% in the final cohort. According to the conventional rule of thumb (10–15 outcome events per predictor), approximately 70–105 RIP events, corresponding to a total sample size of roughly 140–210 patients, are required for a fully powered multivariable model. Consequently, the findings of this study should be interpreted with caution and warrant validation in larger, prospective cohorts with adequate statistical power.

Additionally, the absence of a validation cohort may hinder our ability to fully elucidate the findings, highlighting the need for further research in this field. Potential confounding factors related to baseline oral health may have also influenced the observed associations. Although all participants underwent standardized professional dental cleaning before CCRT, interindividual differences in plaque control, gingival inflammation, and adherence to oral hygiene instructions could have impacted the risk of periodontal breakdown during and after therapy. Such variations may modulate local inflammatory responses, alter biofilm composition, and affect mucosal or connective-tissue resilience. While these factors were minimized through uniform pre-treatment dental management, they remain an inherent limitation of retrospective analyses and should be considered in future prospective trials.

Regarding excluded patients, although detailed comparisons were not feasible, they appeared broadly similar in age and tumor stage distribution to those analyzed. However, they included a higher proportion of edentulous individuals and patients without baseline dental evaluations. Since these exclusions primarily reflect the availability of oral health documentation rather than oncologic factors, some degree of selection bias is possible, thereby limiting the generalizability of our findings to dentate patients with complete periodontal assessments.

It is also essential to acknowledge that our study analyzed data collected only on the first day of CCRT treatment. Therefore, the current CAR cutoff may not accurately identify the optimal threshold for categorizing LA-HNC risk, as levels of ALB and CRP can fluctuate significantly during and after concurrent CCRT. Furthermore, we may have overlooked the potential to establish reliable cause-and-effect relationships between cohorts with elevated CARWL scores and cytokine/chemokine levels, nutritional status, and immune-inflammatory markers such as IL-1, IL-6, and TNF-α. Consequently, the findings of this study should be regarded as exploratory and hypothesis-generating rather than definitive recommendations, until additional well-designed, large-scale research studies addressing these important themes provide supporting data.

Despite these limitations, the components of the CARWL index are easily accessible, simple to calculate, cost-efficient, and consistently exhibit specific characteristics, making the index a practical biomarker for frequent clinical use. Therefore, if further research confirms its effectiveness, the newly developed CARWL score could categorize LA-HNC patients into risk groups based on their likelihood of RIP. This could facilitate careful monitoring of individuals at high risk and enable the early implementation of preventive measures against periodontitis in its initial stages.

## Conclusion

RIP is a significant complication that can arise during the treatment of LA-HNC patients, carrying poor prognostic implications, including an increased risk of tooth loss, osteoradionecrosis, and WL. Our research indicates that the newly developed CARWL index serves as a reliable biomarker for predicting the occurrence rates of RIP in patients with LA-HNC. If future research corroborates the results outlined in this study, this biological marker could represent a breakthrough in identifying high-risk individuals, potentially enhancing current methods and leading to the development of effective preventive strategies and post-assessment protocols.

**Consent for publication:** All patients, or their legally authorized representatives, provided informed consent prior to the evaluation. This consent covered the acquisition and analysis of sociodemographic, dental, and medical records, as well as the collection of blood samples and the publication of the results.

## Supplemental data

## Data Availability

Data ownership and storage are maintained by the Baskent University Medical Faculty, which prohibits public sharing. However, researchers who meet the criteria for accessing confidential data may request access through the Baskent University Institutional Data Access / Ethics Committee. For inquiries, please contact the Baskent University Ethics Committee at adanabaskent@baskent.edu.tr.
